# Patients Hospitalized in General Wards via the Emergency Department: Early Identification of Predisposing Factors for Death or Unexpected Intensive Care Unit Admission—A Historical Prospective

**DOI:** 10.1155/2014/203747

**Published:** 2014-01-29

**Authors:** Thierry Boulain, Isabelle Runge, Nathalie Delorme, Angèle Bouju, Antoine Valéry

**Affiliations:** ^1^Medical-Surgical Intensive Care Unit, Hôpital de La Source, Centre Hospitalier Regional d'Orléans, BP 6709, 45067 Orléans, France; ^2^Adult Emergency Department, Hôpital de La Source, Centre Hospitalier Regional d'Orléans, 45067 Orléans, France; ^3^Hospital Medical Information Department, Hôpital de La Source, Centre Hospitalier Regional d'Orléans, 45067 Orléans, France

## Abstract

*Background.* To identify, upon emergency department (ED) admission, predictors of unexpected death or unplanned intensive care/high dependency units (ICU/HDU) admission during the first 15 days of hospitalization on regular wards. *Methods.* Prospective cohort study in a medical-surgical adult ED in a teaching hospital, including consecutive patients hospitalized on regular wards after ED visit, and identification of predictors by logistic regression and Cox proportional hazards model. *Results.* Among 4,619 included patients, 77 (1.67%) target events were observed: 32 unexpected deaths and 45 unplanned transfers to an ICU/HDU. We identified 9 *predictors of the target event* including the oxygen administration on the ED, unknown current medications, and use of psychoactive drug(s). All predictors put the patients at risk during the first 15 days of hospitalization. A logistic model for *hospital mortality prediction* (death of all causes) still comprised oxygen administration on the ED, unknown current medications, and the use of psychoactive drug(s) as risk factors. *Conclusion.* The “use of oxygen therapy on the ED,” the “current use of psychoactive drug(s)”, and the “lack of knowledge of current medications taken by the patients” were important predisposing factors to severe adverse events during the 15 days of hospitalization on regular wards following the ED visit.

## 1. Introduction

Clinical deterioration leading to unexpected death or intensive care unit (ICU) admission can occur during the course of patients hospitalized on regular wards. These unexpected events are often not only explained by the natural course of the patients' diseases but can also result from several dysfunctions of the care system [1–3], source of enormous human and financial impact [4, 5]. As they may be avoided [1, 6], several health care systems have developed organizational measures to prevent them. The most topical measure is the implementation of the so-called “Patient At Risk Team,” “Critical Care Outreach Team”, or “Medical Emergency Team” [7]. However, this approach has not definitively proved its effectiveness in lives and cost saving [7–9]. Another way to prevent adverse events due to late recognition of clinical deterioration may be to admit fragile and at-risk patients to intermediate care settings (high dependency units (HDU)). Nevertheless, these units are staff and cost consuming and mostly comprise only few beds [10]. Usually, patients presenting with obvious or suspected organ failure at the emergency department (ED) are transferred to the ICU or HDU. For the remaining patients needing hospitalization, the choice of the setting depends on the type of the patient's illness, the available number of HDU beds, and also the physician's ability to recognize patients at the higher risk of deterioration. At the end of this decision process, some patients at high risk of clinical worsening may be transferred to the ward. Some of them will experience unexpected death or delayed ICU admission [11]. Numerous attempts were made in cohorts of ED patients to find relevant predictors of clinical deterioration and to include them into a predictive score. Nevertheless, only subpopulations of patients are reported [12–16], often excluding surgical patients. Further, including both patients discharged home and patients presenting with frank distress (often needing immediate ICU admission) [17–20] may have strengthened the global performance of death predicting scores but leaves the outcome of the numerous intermediate-acuity patients difficult to predict [20]. Additionally, most of the predictive scores have focused on vital signs without taking into account laboratory data or past medical history, despite the fact that these data impact the decision-making process.

We hypothesized that past medical history, physiological variables, and routine laboratory variables could be of interest to better predict clinical deterioration during hospitalization on regular wards. The primary objective of our work was to search, during the first 6 hours of ED admission, for demographic, clinical, and also laboratory variables linked to death that did not result from do-not-resuscitate orders or unplanned ICU/HDU admission during the first 15 days of hospitalization on regular medical or surgical wards.

## 2. Methods

### 2.1. Setting

This prospective cohort study was conducted in our regional 1000-bed hospital in Orléans (France), in the medical-surgical adult ED (the pediatric and the obstetric/gynecology EDs did not participate). The hospital comprises four intensive or intermediate care units for adult patients: neurosurgical ICU (4 beds), general surgical ICU (8 beds), coronary intensive care unit (10 beds), medical HDU (13 beds), and medical-surgical ICU (18 beds).

The Ethics' Committee of *Association des Réanimateurs du Centre-Ouest* approved the study and allowed to waive patients' consent. Patients admitted to the ED were informed, as routinely done, that (1) observational studies could be done using data related to their hospitalization and (2) they had the right to refuse to be enrolled in such studies.

### 2.2. Study Design and Selection of Participants

All consecutive patients aged 15 to 90 years and admitted to the adult ED before admittance to surgical or medical regular wards were include. We did not included patients who died within the ED, who were discharged home or directly transferred to ICU or HDU (without general ward admission), or for whom a do-not-resuscitate order was already known. Because of the rarity of unexpected deaths observed in gynecologic/obstetric, pediatric, geriatric, and orthopedic wards (in 2003 and 2004 analysis), patients transferred to these settings were not included.

### 2.3. Data Collection

For each included patient, the following clinical, laboratory, and demographic data were prospectively recorded, on a daily basis by review of patient's ED charts routinely filled in by ED nurses and physicians: date and time of admission; gender; age; unknown underlying diseases and past medical history at the time of ED visit; palliative care either underway or decided during ED visit; obvious or probable chronic alcoholism; known chronic obstructive pulmonary disease (COPD); presence of other respiratory chronic diseases; solid cancer considered not definitively cured; ongoing hematological malignancy; chronic renal insufficiency (serum creatinine > 250 *μ*Mol/L); chronic haemodialysis or peritoneal dialysis; chronic arterial hypertension; coronaropathy; type I or type II diabetes mellitus; past hospitalization for psychiatric disorder or for suicide attempt; known cerebral illness (dementia, degenerative encephalopathy, and epilepsy); knowledge of current medications; known treatment by corticosteroids, nonsteroidal anti-inflammatory drugs (NSAID), antiplatelet drugs, angiotensin-converting enzyme inhibitors (ACEI) or angiotensin receptor blockers (ARB), diuretics, oral anticoagulant, psychoactive drugs (benzodiazepines, neuroleptics, antidepressors, anticonvulsants, morphinomimetics, tramadol, and lithium); admission for possible bacterial infection (recent fever and/or antibiotics prescribed before or during ED admission); admission for trauma; admission for acute, voluntary or not, intoxication (ethanol, illicit drugs, psychoactive drugs, smoke, plants, and agricultural products); acute confusion observed on the ED; witnessed seizures before or during ED stay; administration of oxygen therapy on the ED; minimal and maximal values (recorded during the first 6 hours in the ED) of blood pressure, heart rate, respiratory rate, capillary blood glucose level, and body temperature; maximal score on Glasgow coma scale; minimal oxygen saturation measured by pulse oximetry (SpO_2_). Laboratory variables recorded, when available on the ED, were ionogram (natremia, serum chloride, kaliemia, alkaline reserve, and blood protein level); serum creatinine; blood cell count; hemoglobin; prothrombin time (expressed as international normalized ratio (INR)); activated partial thromboplastin time (aPTT); liver aminotransferases (AST and ALT); total bilirubin.

We also collected additional variables: admission “at night” (between 19:00 and 07:00); admission on weekend (from 19:00 on Friday to 07:00 on Monday); presence of a systemic inflammatory response syndrome (SIRS) [25] during the ED stay; anion gap (calculated as [Na^+^ + K^+^] − [Cl^−^ + alkaline reserve]) when blood ionogram was available. Categorical variables were coded as 0 (absence) or 1 (presence) and were entered on a computerized spreadsheet.

Of note, for each patient admitted to the ED, the emergency physician systematically searched for previous hospitalization, known illnesses, and medications taken in the electronic hospital registry available at the ED. The item “lack of knowledge of the usual medications or the chronic illnesses” was ticked only after unsuccessful search on electronic hospital registry and after not informative interview of patient, familial practitioner or next of kin.

### 2.4. Missing Data

We planned to only analyze clinical and laboratory variables recorded in more than 85% of the patients. Among these parameters, missing values were recorded as “normal” for clinical items or replaced by the mean value in the whole population for laboratory items.

### 2.5. Definition and Recording of the Target Event

Once a week, two investigators (ND and AB) recorded the occurrence of death or unplanned admission in an intensive or intermediate care unit during the first 15 days of hospitalization on regular wards, as provided by our Hospital Medical Information Department. Deaths that occurred on the wards were classified as unexpected deaths or deaths with do-not-resuscitate order after review of patient's chart and after interview of the attending nursing and medical staff. *The chosen target event was a composite end-point: unplanned death or unplanned admission to ICU or HDU, during the first 15 days of admission on the ward*.

### 2.6. Calculation of Sample Size

As the frequency of the target event is low (based on our hospital statistics for 2003 and 2004 and also based on a study by Bristow et al. [11]), around 1.7%, a 7-month period of study was deemed necessary to observe 50 to 80 target events.

### 2.7. Data Analysis

The association between each of the recorded variables and the occurrence of the target event (unplanned death or unplanned admission to ICU or HDU) was examined by Fisher's exact test or by Student's *t*-test when appropriate. The variables linked to the target event with *P* < 0.15 were checked for colinearity: when 2 variables belonging to the same category of data (i.e., either medications, demographic data, chronic illnesses, on clinical or biological symptoms at ED admission) were correlated with an absolute value of Pearson's correlation coefficient *r* ≥ 0.15, only the variable linked to the target event with the smallest *P* value was kept for further analysis. We also examined the correlation coefficients between existing chronic illnesses and specific taken medications (e.g., chronic hypertension and treatment by diuretics or beta-blockers) and between the administration of oxygen during the ED stay and the respiratory rate and SpO_2_. When two variables were correlated with *r* ≥ 0.15, the variable with the less number of missing values was kept for further analysis. All selected variables were then entered into a logistic regression model also to examine their relationship with time, in a Cox proportional hazards model, both with stepwise backward procedure.

Continuous variables were introduced after transformation in categorical variables when their distribution was not normal. Variables that remained linked to the target event with *P* < 0.05 after multivariate analysis were considered as possible predictors of the target event.

The discriminative power of the logistic regression model was assessed by calculating the area under the receiver operating characteristics curve (AUROC) [26] and by testing calibration (i.e., the degree of correspondence between predicted and observed target event rate) by the Hosmer-Lemeshow *χ* ^2^ test [27]. Our multivariate logistic regression analysis was made with and also without incorporating biological variables (in order to assess the improvement they allow in performance).

At last, we reran the logistic analysis to build a model for hospital mortality prediction (all causes of death) and compared its performance (AUROC) to the performance of several published predictive scores applied to our data set [28].

Values were expressed as the mean ± standard deviation (SD) (continuous variables), unless otherwise specified, or as a percentage of the group from which they were derived (categorical variables). Adjusted odds ratios (OR) and hazard ratios (HR) are provided with their 95% confidence interval (95% CI). All *P* values were two-tailed, and *P* < 0.05 indicates statistical significance. Descriptive statistics and multivariate analyses were performed with MedCalc 10.3.2.0 (Mariakerke, Belgium).

## 3. Results

**Figure 1:**
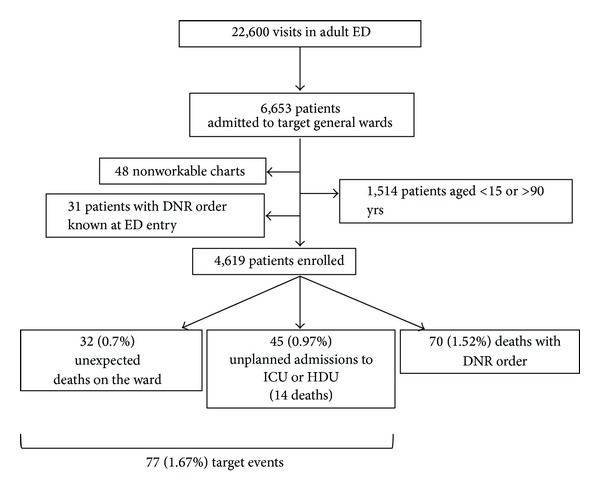
Flow diagram. ICU: intensive care unit; HDU: high dependency units; ED: emergency department; DNR order: do-not-resuscitate order.

From February 12, to September 12, 2005, 4,619 patients were included (Figure 1).

Demographic, clinical, and biological data are summarized in Table 1. Past medical history and current medications taken could not be retrieved during the ED stay for 4.3% and 10.7% of the cases, respectively.

A 2% sample (92 patients' charts) was randomly extracted from the dataset to check for reliability. Overall, 97.5% (5,382/5,520) of the computerized items for these patients agreed with the original written records.

Among the 4,619 enrolled patients, 77 (1.67%) target events were observed: 32 (0.7%) unexpected deaths in the ward and 45 (0.97%) admissions in ICU or HDU (14 deaths). During the first 15 days, 70 (1.52%) deaths after do-not-resuscitate order occurred.

The 15-day mortality and the hospital mortality rates were 2.51% and 4.2%, respectively.

### 3.1. Prediction of the Target Event

Thirty-five variables were linked to the target event (occurrence of unplanned death or unplanned admission to ICU or HDU, during the first 15 days of admission on the ward) with *P* < 0.15 in univariate analysis (Table 2). Capillary blood glucose level, minimal SpO_2_, and respiratory rate were recorded in only 78.2%, 70.4%, and 62.2% of the cases, respectively, and, as planned, were not used for further analysis because they were strongly correlated with other variables with no missing value. In the 3610 patients with recorded capillary blood glucose level, the variable “diabetes mellitus” was strongly correlated with capillary blood glucose level (*P* < 0.0001; *r* ^2^ = 0.20). In the 2874 patients with recorded respiratory rate, the variable “oxygen administration in the ED” was strongly correlated with maximal respiratory rate (*P* < 0.0001; *r* ^2^ = 0.15) and, in the 3252 patients with recorded minimal SpO_2_, the variable “oxygen administration in the ED” was strongly correlated with maximal respiratory rate (*P* < 0.0001; *r* ^2^ = 0.17).

**Figure 2:**
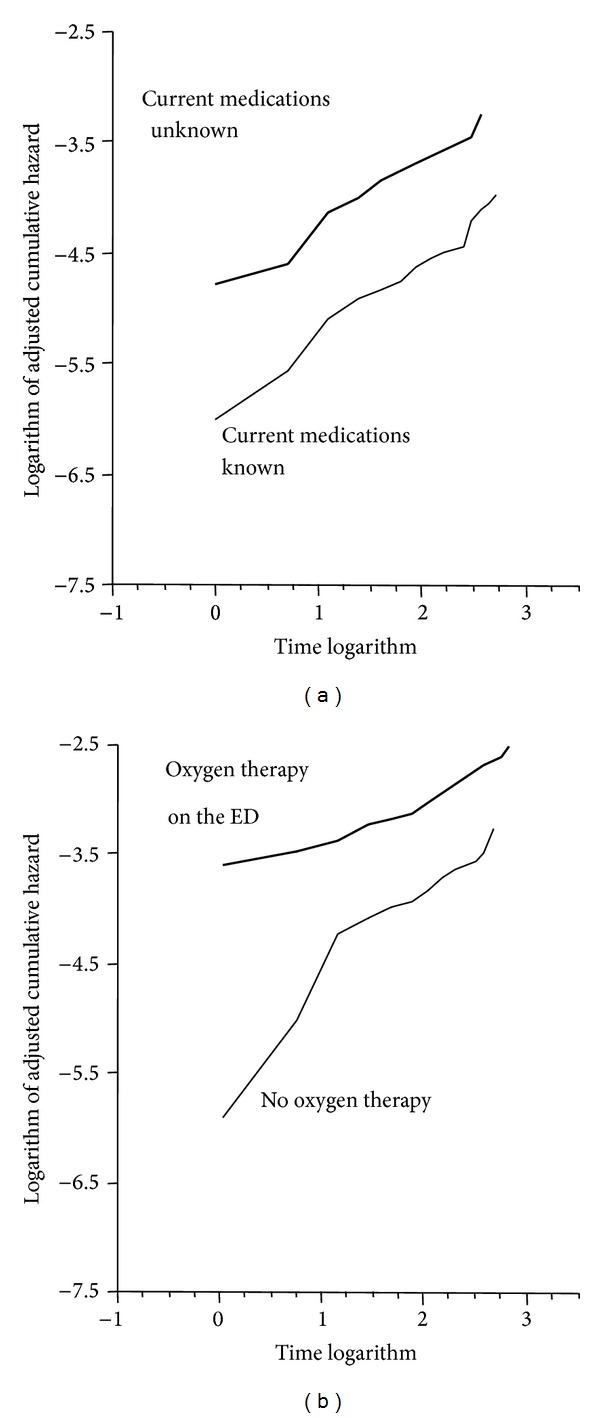
Cumulative hazard due to the variables “current medications unknown” and “oxygen therapy on the ED” plots the logarithm of the cumulative target event rate against the logarithm of time according to the presence or not of the variables current medications unknown (a) and oxygen therapy in the ED (b). It permits graphically verifying that the hazard ratios are independent of time approximately parallel curves. This was also examined for all other variables retained in the Cox model to ascertain that the assumption of hazard proportionality was not violated. ED: emergency department.

After planned elimination of some other variables due to colinearity and after recoding the variables “age”, “minimal systolic blood pressure”, “platelet count”, “serum chloride,” and “anion gap”, which had a skewed distribution, 19 variables were kept for further analysis. Among them, the following 9 remained significantly linked to the target event (*P* < 0.05) either after adjustment by multivariate logistic regression or by Cox proportional hazards model (Table 3): systolic arterial pressure below 110 mmHg, platelet count below 150 G/L, serum chloride below 100 mmol/L, male gender, known chronic hypertension, oxygen administration on the ED, unknown current medications, use of psychoactive drug(s), and the presence of at least 3 SIRS items. The time independency of hazards ratios of the Cox model was verified graphically for all predictors, as illustrated in Figure 2 for the variables “unknown current medications” and “oxygen therapy on the ED.”

The target event probability derived from the logistic model had reasonable discriminative power (AUROC = 0.80 (95% CI: 0.79 to 0.81)) and was well calibrated (Hosmer-Lemeshow *χ* ^2^ = 6.31; *P* = 0.61). It performed better (*P* < 0.05) than a similar model not incorporating laboratory data that retained 6 predictive factors (age, male gender, chronic hypertension, unknown current medications, use of psychoactive drugs, and oxygen therapy in the ED (odds ratios not shown)) and had an AUROC of 0.75 (0.74–0.77).

### 3.2. Prediction of Hospital Mortality

Our final logistic model for hospital mortality prediction that retained 13 variables (Table 4) was associated with good discriminatory power (AUROC = 0.87 (95% CI: 0.86 to 0.88)) and was well calibrated (Hosmer-Lemeshow *χ* ^2^ = 7.73; *P* = 0.46). Of note, the 13 retained factors still comprised the variables “use of oxygen therapy in the ED”, “unknown current medications,” and “use of psychoactive drugs”. Our model performed better than the 5 previously published predictive scores [12, 21–24] (*P* < 0.0001 for all 5 comparisons) (Table 5).

The logistic model for hospital mortality prediction not incorporating the biological variables comprised 7 variables (still comprising the variables “use of oxygen therapy in the ED”, “unknown current medications,” and “use of psychoactive drugs”) and had an AUROC of 0.82 (95% CI: 0.81 to 0.83), that is, less performing than the 13-variable model (*P* = 0.034).

## 4. Discussion

The main finding of our study is the identification of new factors predisposing patients with intermediate severity of illness to clinical deterioration and even to death while hospitalized on regular wards via the ED: the initiation of oxygen therapy in the ED, the use of psychoactive drug(s), and the lack of knowledge of medications regularly taken by the patient. Additionally, our work underlines the limits of validated predictive models [22, 23], underscoring the improvement allowed by the incorporation of patient's past medical history and laboratory variables into a predictive model as ours.

In our study patients, the target event rate was 1.7%, that is, similar to that reported elsewhere [11] and the hospital mortality rate was 4.2%. The latter is difficult to compare to published data because of heterogeneity of ED cohorts in terms of inclusion criteria (e.g., inclusion or not of patients discharged home or rapidly transferred to ICU) and observation period.

### 4.1. Clinical and Laboratory Predictors

In our study patients, the respiratory rate and the SpO_2_ have been recorded on ED charts in only 62% and 70% of the cases, respectively, in our study, like it is often not recorded in many hospitals [29]. As planned, we examined the initiation of oxygen therapy on the ED as an important variable, reflecting poor respiratory condition, which could be seen as a surrogate for respiratory rate. Indeed, oxygen administration was strongly linked to the respiratory rate and to the SpO_2_ and appeared in our study as one of the most important variables to consider. Moreover, SpO_2_ could be considered as quite imprecise as concomitant fractional concentration of inspired oxygen was not recorded in our study, as also often observed in others [30]. The same limitations might apply to the respiratory rate.

The regular and current use of psychoactive drugs was a significant factor for clinical deterioration during hospitalization in our study cohort. This is in line with previous studies [31] and is probably explained by the increased risk of various pathologic events associated with psychoactive drugs use, such as delirium, withdrawal syndromes, aspiration pneumonia, muscles weakness, falls, and consciousness impairment. However, we cannot exclude that the role of psychoactive drugs might not be found in other countries, as the use of these drugs was very frequent in our study population (21.5%), as it is generally the case in the French population [32].

Interestingly, our analysis disclosed that the lack of knowledge of the medications regularly taken by the patients (10.7% of the cases) is a risk factor for further clinical deterioration. This may be not only due to patients' inability to give the information but also due to the fact that it could not be found in the hospital electronic database, the absence of next-of-kin, or the impossibility to reach the familial practitioner. Our findings underline the paramount importance of meticulous search for this information at ED presentation, in order to avoid the ignorance of chronic organ failure, to prevent drug withdrawal syndromes in patients taking psychoactive drugs, or to avoid adverse drug interactions [33]. Interestingly, despite the fact that the list of the current medications is often retrieved during the first days of hospitalization, our Cox analysis tends to show that the lack of this information while on the ED put the patients at risk even during the following 15 days, suggesting that it may be of utmost importance to get this information earlier, that is, during the ED stay.

As in other studies [13, 22, 30, 34], important physiological variables such as heart rate or the level of consciousness were not retained in our analyses. Among other reasons, this could be due to the fact that we excluded patients with obvious distress and urgent need of intensive care referral.

### 4.2. Strengths and Limitations

To our knowledge, although our data are now 8 years old, our work is the first study that focused on the risk factors for hospital death or delayed ICU/HDU referral upon ED admission of unselected (i.e., medical and surgical) patients admitted to the ward via the ED. Indeed, numerous studies have been performed in selected populations of patients with trauma [35, 36], sepsis [15, 16, 20, 37], hypotension [14, 17, 18], nonsurgical patients [12, 13], geriatric patients [38], or only in patients who underwent blood sampling in the ED [39]. However, studies analyzing the whole population of patients admitted to regular wards via the ED are scarce [30, 34]. Of note, as we excluded patients who needed hospitalization on orthopedic or gynecologic/obstetric wards, our study population comprised a large proportion of medical patients. Thus, our cohort may be seen as a selected population. More importantly, unlike most of the above-cited studies, we focused our work on patients with intermediate severity of illness upon ED arrival, that is, excluding those who were discharged home or who had obvious or suspected organ failure that needed rapid ICU or HDU admission. Excluding these patients with very low or very high acuity scores at the ED, while focusing on intermediate-acuity patients, is known to weaken the performance of scores developed to predict death or clinical deterioration [40]. This is the reason why we did not attempt at building and validating another predictive score [30, 34]. Rather, our aim was to identify potential factors that could put the patients of intermediate severity of illness at risk of further deterioration and that could suggest possible avenues for improvement in the care of such patients.

Of note, we focused on the prediction of death that was not the result of palliative care and of unplanned ICU admissions (known to increase the risk of death [41]), in order to, in a pragmatic view, help avoid deaths that should be prevented. To our knowledge, our study is the first work focused on this outcome. This also could be seen as a limitation of our work since differentiating deaths that follow do-not-resuscitate orders or palliative care from actual unexpected deaths is not easy, even after cautious review of medical charts and interviews of the nursing-medical staffs, after which a part of subjectivity could persist. However, the second part of our work focusing on the prediction of hospital death (of any cause) tends to show that the most important variables retained in our primary analysis were not the result of bias as “oxygen therapy on the ED”, “use of psychoactive drug(s)”, and “unknown current medications” still remain linked to hospital death.

The high amount of missing data in our work, despite reflecting the real-life conditions in which it was performed, could have weakened the power of our multivariate analyses. However, we do not believe it could have biased our identification of risk factors as the incidence of the target event was consistently far less in patients with no respiratory rate measured at the ED (0.92%) or no SpO_2_ measured (0.73%), for example, than in patients with complete data.

Finally, our data are now old data, and despite the absence of obvious advances in the care of emergency department patients in our hospital since we conducted our study (same hospital electronic record, no automatic connection to the patient record that might be owned by the familial practitioner, and no increase in medical staff in the emergency department), our results might not be replicable today. For this reason, we plan to repeat the study in the near future and we are working to get closer to other hospitals in our region, with the aim to conduct a multicenter study that could give results with better external validity.

## 5. Conclusion

Our work identified the “use of oxygen therapy on the ED”, the “current use of psychoactive drug(s)”, and the “lack of knowledge of current medications” as important factors that predispose patients to severe clinical deterioration during the 15 days of hospitalization on regular wards that follow ED visit.

## Figures and Tables

**Table 1:** Demographic characteristics of 4,619 consecutive patients admitted to the emergency department and then hospitalized on regular wards.

	Entire cohort *n* = 4,619 (%)
Age, yrs (mean ± SD)	55.2 ± 21
Gender, male/female, in %	54%/46%
Surgical patients* (%)	342 (7.4)
Medical patients (%)	4277 (92.6)
First hospitalization ward after ED visit	
Digestive and thoracic surgery	493 (10.7)
Neurosurgery	64 (1.4)
Other surgical wards	385 (8.3)
Short-stay ward	1654 (35.8)
Infectious diseases ward	402 (8.7)
Hepatogastroenterology	258 (5.6)
Pneumology	246 (5.3)
Cardiology	234 (5.1)
Neurology	228 (4.9)
Other medical wards	655 (14.2)
Major diagnostic categories as coded in hospital database*	
Nervous system	614 (13.3)
Eye	50 (1.1)
Ear, nose, mouth, and throat	68 (1.5)
Respiratory system	537 (11.6)
Circulatory system	366 (7.9)
Digestive system	559 (12.1)
Hepatobiliary system and pancreas	203 (4.4)
Musculoskeletal system and connective tissues	184 (4%)
Skin, subcutaneous tissue, and breast	159 (3.4)
Endocrine, nutritional, and metabolic system	96 (2.1)
Kidney and urinary tract	297 (6.4)
Male and female reproductive system	106 (2.2)
Blood and myeloproliferative diseases	114 (2.4)
Infectious and parasitic diseases and HIV infection	119 (2.6)
Mental diseases and disorders	92 (2)
Alcohol/drug use induced disorders	536 (11.6)
Injuries, poison, and toxic effect of drugs	180 (3.9)
Others	344 (7.5)
Hospital mortality rate	195 (4.2)
Destination of survivors after hospitalization	
Home	4088 (88.5)
Rehabilitation centre	199 (4.3)
Long-term care	16 (0.3)
Psychiatric hospital	121 (2.6)

*We based disease presence and diagnostic history on the presence of ICD-10 (international classification of diseases, 10th revision) codes; ED: emergency department; HIV: human immunodeficiency virus.

**Table 2:** Clinical and biological variables linked to the target event (death or unplanned ICU or HDU admission during the first 15 days of hospitalization).

	Missing values (%)	Patients with target event (*n* = 77)	Patients free of target event (*n* = 4542)	*P* value^c^
Categorical variables				
Male gender	0	53 (68.8%)	2443 (53.8%)	0.0107
Underlying diseases and past medical unknown history	0	6 (7.8%)	194 (4.3%)	0.147
COPD	0	13 (16.9%)	375 (8.3%)	0.012
Solid unresolved cancer	0	1 (1.3%)	291 (6.4%)	0.0916
Chronic arterial hypertension	0	30 (39%)	954 (21%)	0.0004
Coronaropathy	0	12 (15.6%)	460 (10.1%)	0.1268
Diabetes mellitus type II	0	13 (16.9%)	364 (7.7%)	0.0082
Diabetes mellitus type I or II	0	16 (20.8%)	592 (12.7%)	0.0558
Unknown current medications	0	16 (20.8%)	473 (10.4%)	0.0075
Beta-blockers	0	16 (20.8%)	506 (11.1%)	0.0162
Diuretics	0	16 (20.8%)	588 (12.9%)	0.0582
Oral anticoagulant	0	10 (13%)	368 (8.1%)	0.1375
NSAID	0	18 (23.4%)	635 (14%)	0.03
Psychoactive drugs	0	31 (40.3%)	1459 (32.1%)	0.1404
Admission for possible bacterial infection^a^	0	27 (37.1%)	1064 (23.4%)	0.0212
Admission for acute intoxication	0	1 (1.3%)	646 (14.2%)	0.0002
Oxygen administration during the ED stay	0	36 (46.8%)	742 (16.3%)	<0.0001
Presence of SIRS^b^ during the ED stay	0	51 (53.7%)	953 (31.8%)	<0.0001
Continuous variables				
Age (yrs)	0	66.6 ± 16.6	55 ± 21.1	<0.0001
Minimal heart rate (beats/min)	0	85 ± 17	82 ± 18	0.1323
Maximal heart rate (beats/min)	0	96 ± 20	89 ± 19	0.0029
Minimal systolic blood pressure (mmHg)	0	125 ± 29	131 ± 26	0.0258
Minimal diastolic blood pressure (mmHg)	0	68 ± 19	71 ± 16	0.1042
Minimal body temperature (°C)	0	36.7 ± 0.74	36.9 ± 0.79	0.111
Capillary blood glucose level (g/L)	21.8	1.51 ± 0.76 (*n* = 65)	1.26 ± 0.6 (*n* = 3545)	0.0007
Minimal SpO_2_ (%)	29.6	93 ± 7 (*n* = 67)	96 ± 4 (*n* = 3185)	<0.0001
Minimal respiratory rate (cycles/min)	37.8	22 ± 5 (*n* = 61)	20 ± 5 (*n* = 2813)	0.04
Maximal respiratory rate (cycles/min)	37.8	26 ± 9 (*n* = 61)	22 ± 6 (*n* = 2813)	<0.0001
Platelet count (G/L)	12.2	200 ± 98 (*n* = 74)	230 ± 88 (*n* = 3981)	0.0041
Neutrophils count (G/L)	12.2	8.4 ± 4.8 (*n* = 74)	7.1 ± 5.9 (*n* = 3981)	0.056
Prothrombin time (INR)	13.2	1.75 ± 1.7 (*n* = 70)	1.23 ± 0.72 (*n* = 3938)	<0.0001
Serum chloride (mEq/L)	13.6	99.8 ± 7.3 (*n* = 77)	101.7 ± 5.5 (*n* = 3914)	0.0027
Blood protein level (g/L)	13.6	68.7 ± 7.4 (*n* = 77)	70 ± 7.5 (*n* = 3914)	0.1312
Serum creatinine (*μ*mol/L)	13.6	126 ± 108 (*n* = 77)	99 ± 80 (*n* = 3914)	0.0097
Anion gap (mEq/L)	13.6	14.2 ± 4.5 (*n* = 77)	13.1 ± 3.7 (*n* = 3914)	0.0077

^a^Admission for possible bacterial infection: recent fever and/or antibiotics prescribed before or during ED admission. ^b^As respiratory rate was not recorded in 37.8% of the cases and leukocytes count was not performed in 12.2% of the cases, the incidence of SIRS was underestimated; ^c^categorical variables were compared by Fisher's exact test and continuous variables by unpaired two-tailed Student's *t*-test.

ICU: intensive care unit; HDU: high dependency unit; COPD: chronic obstructive pulmonary disease; ACEI: angiotensin-converting enzyme inhibitors; ARB: angiotensin receptor blockers; ED: emergency department; NSAID: nonsteroidal anti-inflammatory drug; SIRS: systemic inflammatory response syndrome; SpO_2_: oxygen saturation measured by pulse oximetry.

**Table 3:** Variables identified as potential predisposing factors for 15-day clinical worsening during hospitalization on regular wards, by multivariate logistic regression and by Cox proportional hazards models.

Predisposing factor	Logistic regression^a^			Cox model^b^		
	Adjusted odd ratio	95% CI	*P*-value	Adjusted hazard ratio	95% CI	*P*-value
Unknown current medications	2.96	1.58–5.54	0.0007	3.15	1.72–5.76	0.0002
Oxygen administration in the ED	2.86	1.74–4.70	<0.0001	2.31	1.43–3.73	0.0007
Serum chloride below 100 mmol/L	2.32	1.45–3.72	0.0005	1.92	1.22–3.03	0.0053
Chronic hypertension	2.24	1.37–3.67	0.0013	1.75	1.09–2.80	0.0204
Presence of at least 3 SIRS items	2.05	1.19–3.54	0.0099	1.76	1.05–2.94	0.0339
Platelet count below 150 g/L	2.03	1.17–3.50	0.0113	1.73	1.03–2.92	0.0401
Male gender	1.92	1.15–3.13	0.0123	1.92	1.08–2.86	0.0247
Systolic arterial pressure below 110 mmHg	1.86	1.14–3.06	0.0138	1.97	1.23–3.16	0.0051
Use of psychoactive drug(s)	1.66	1.00–2.77	0.0499	1.70	1.03–2.81	0.0380
Solid unresolved cancer*	0.09	0.01–0.66	0.0178	0.09	0.01–0.68	0.0200

ED: emergency department; CI: confidence interval; SIRS: systemic inflammatory response syndrome (ref. bone).

^a^Overall model fit: log likelihood chi-square = 109 (*P* < 0.0001). ^b^Overall model fit: log likelihood chi-square = 86 (*P* < 0.0001). *A 10th predictor in addition to the nine cited in the text was identified. The presence of unresolved cancer appeared as protective against target event occurrence in our cohort. However, the 95% CI of odd-ratio and hazard ratio were very large because only one target event occurred in the 291 unresolved cancer patients. This is due to the fact that we did not consider deaths that resulted from do-not-resuscitate orders as target events, whereas this is a frequent mode of death in cancer patients. As this could appear as counterintuitive, we did not cite the variable “unresolved cancer” in our results section. As this may create instability in our model, we reran the same analysis without incorporating the variable “unresolved cancer.” Both models (logistic and Cox regressions) kept a good overall fit (*P* < 0.0001) with the same covariates (except for “use of psychoactive drugs” that no longer reached statistical significance), and the logistic model kept a rather good AUROC value: 0.78 (0.77–0.80). In both analyses, the variables “unknown taken medications” and “oxygen therapy in the ED” remained the most powerful predictors (odd ratio 2.7 and 2.8, resp.; hazard ratio 2.8 and 2.3, resp.).

**Table 4:** Factors retained by multivariate logistic regression to predict hospital death (of all causes) in our population.

Variable	Adjusted odd ratio	95% CI	*P*
Glasgow coma score below 12	18.02	7.66–42.41	<0.0001
Age higher than 60 yrs	3.90	2.47–6.20	<0.0001
Oxygen therapy on the ED	2.22	1.53–3.21	<0.0001
Platelet count below 150 g/L	2.13	1.40–3.25	0.0004
Serum chloride below 100 mMol/L	2.08	1.45–2.96	0.0001
Use of psychoactive drug(s)	2.02	1.39–2.94	0.0003
Current medications unknown	1.89	1.11–3.23	0.0188
Kaliemia (for 1 mMol/L increase)	1.56	1.23–1.97	0.0002
Male gender	1.47	1.02–2.13	0.0380
INR (for 1 unit increase)	1.22	1.07–1.39	0.0035
Neutrophils (for 1 g/L increase)	1.06	1.03–1.09	<0.0001
Maximal heart rate (for 1 beat/min increase)	1.01	1.00–1.02	0.0022
Hemoglobin (for 1 g/dL increase)	0.85	0.79–0.92	<0.0001

ED: emergency department; INR: international normalized ratio for prothrombin time.

**Table 5:** Comparison of current logistic model with existing scores for the prediction of hospital death.

	AUROC	95% CI	*P* for comparison with current model
Current model	0.860	0.846–0.874	—
MEWS [21]	0.675	0.655–0.694	<0.001
Goodacre's score [22]	0.766	0.748–0.783	<0.001
WPSS [23]	0.610	0.590–0.630	<0.001
REMS [12]	0.740	0.722–0.757	<0.001
RAPS [24]	0.687	0.668–0.706	<0.001

AUROC: area under the receiver operating characteristics curve; CI: confidence interval. We applied the calculation of five published scores developed to predict hospital death in our patients with complete data (2367 (51% of our data set) including 126 hospital deaths (5.3%)) for these calculations: modified early warning system (MEWS) [21], Goodacre's score [22], worthing physiological scoring system (WPSS) [23], rapid emergency medicine score (REMS) [12], and rapid acute physiology score (RAPS) [24]. Of note, these scores were not initially developed to predict only death not resulting from do-not-resuscitate order.

## Abbreviations

ACEI: Angiotensin-converting enzyme inhibitors
ALT: Alanine aminotransferase
aPTT: Activated partial thromboplastin time
AR: Angiotensin receptor blockers
AST: Aspartate aminotransferase
AUROC: Area under the receiver operating characteristics curve
CI: Confidence interval
COPD: Chronic obstructive pulmonary disease
ED: Emergency department
HDU: High dependency unit
HIV: Human immunodeficiency department
HR: Hazard ratio
ICD-10: International classification of diseases, 10th revision
ICU: Intensive care unit
INR: International normalized ratio
MEWS: Modified early warning system
NSAID: Nonsteroidal anti-inflammatory drugs
OR: Odds ratio
RAPS: Rapid acute physiology score
REMS: Rapid emergency medicine score
SD: Standard deviation
SIRS: Systemic inflammatory response syndrome
SpO_2_: Oxygen saturation measured by pulse oxymetry
WPSS: Worthing physiological scoring system.
